# Editorial: Trends in cotton breeding: Meeting the challenges of the 21st century

**DOI:** 10.3389/fpls.2022.1019956

**Published:** 2022-10-03

**Authors:** Linghe Zeng, Iain Wilson, Fred M. Bourland

**Affiliations:** ^1^United States Department of Agriculture (USDA), Agriculture Research Service, Crop Genetics Research Unit, Stoneville, MS, United States; ^2^CSIRO, Agriculture and Food, Canberra, ACT, Australia; ^3^University of Arkansas, Northeast Research & Extension Center, Keiser, AR, United States

**Keywords:** cotton breeding, QTL mapping, biotechnology tools, disease, abiotic stress, germplasm

## Introduction

Cotton is one of the most important crops in the world due to its universal use in the textile industry. India, China, the United States, and Brazil produce 75% of the world's cotton. Global cotton production totaled 120.3 million bales in 2021–2022, up 5% from 114.1 million bales in 2020−2021. The United States remains a key cotton exporter, with a total export of 15.5 million bales in 2021. China imported 11 million bales in 2021, which represents a 54.1% increase from 2020.

Global cotton production faces many challenges in the 21st century. Between the rapid increase in human population and the loss of arable land due to soil erosion, soil salinization, harsher climate conditions, and urbanization, the demand for promoting cotton yield is increasing dramatically. Establishing research initiatives to tackle these challenges depends on identifying the major factors that limit increases in yield. These factors have been studied by cotton breeders for decades. They include biotic stresses and abiotic stresses (particularly the water shortage that many regions are experiencing), global climate change, genotype by environmental interactions, limited germplasm resources, and the negative association between yield and fiber quality. The dominance of transgenic cotton has also changed cotton production infrastructures. Throughout the 21st century, cotton breeders have adapted to these changes.

This Research Topic aims to display the perspectives of various disciplines within cotton science and present innovative approaches that can be applied to the science of cotton breeding for genetic improvement of yield and fiber quality. This body of work presents comprehensive reviews and original articles on topics including the history of cotton breeding, biotic and abiotic stresses, germplasm development, and the development of new biotechnological tools for cotton breeding and cultivation.

## Reviews: Germplasm development, breeding, disease and insect resistance

Germplasm collection is an important step in the plant breeding process. Similarly, core collection has become a hot topic in recent years, particularly in terms of the challenges associated with representing genetic diversity with a limited set of germplasm and improving efficiency in the management and utilization of germplasm collection, and in connection with arising problems such as the identification of “real core” and difficulties with the selection of representative accessions. A group of Australian researchers summarized the work by the Commonwealth Scientific and Industrial Research Organization (CSIRO) on cotton germplasm collection and analyzed the value of core collection in cotton breeding (Egan et al.). Their study reviewed the current prospects in the development of core collections and how cotton core collections have played a role in worldwide research discovering new genetic diversity in biotic and abiotic tolerance. The authors expect that core collections will continue to be useful to Australian cotton breeding, especially given that the collections can selectively include traits that fit their industry needs.

Increasing the genetic diversity in cotton germplasm and incorporating new genomic approaches into cotton breeding are critical for meeting 21st-century challenges. Several review articles present the histories and accomplishments of cotton breeding programs in select cotton-producing countries. One Australian group, for example, discussed CSIRO's research activities on improving the genetic diversity of cotton germplasm for yield and fiber quality, pest and disease resistance, and abiotic stress using traditional and genomic approaches (Conaty et al.). The success of CSIRO's cotton breeding programs are the result of Australian cotton breeders' tremendous efforts to battle challenges to cotton production in these areas. The authors of these reviews emphasize the importance of the new breeding methods, i.e., combining phenomics, gene editing, and genomics with host plant resistance, for future success in the development of new germplasm for biotic resistance. Similarly, the increase of plant resistance to insects and disease has been key to reducing the impact of stress (Egan and Stiller).

Glandless cotton offers an attractive protein source for humans and dairy animals because the toxic content, gossypol, has been removed. However, gossypol is a natural insecticide, and its removal leaves glandless cotton vulnerable to plant insects. Moreover, the low yield of glandless cotton leaves it rather unpopular as a planting choice. One article in this series reviewed the research on glandless genetics and the progress in breeding glandless cotton (Zhang and Wedegaertner). The authors note the development of glandless cotton cultivars in New Mexico, US. These cultivars feature improved yield and insect resistance, and thus hold promise for reducing the yield gap with glanded cotton. The authors conclude with an observation on the need to introduce diverse glandless genes into high yielders to further improve yield.

Virus diseases negatively affect cotton yields worldwide. Early detection of viral infection is critical for disease control because its symptoms can be easily confused with those of other conditions such as nutrient deficiency and insect damage. A team of scientists in Brazil presented a review of newly emerging technologies for the detection of viral diseases and their applications in cotton breeding (Tarazi and Vaslin). Serological and molecular virus-detecting techniques can detect virus in plants and identify resistant lines. However, as the authors point out, although molecular detecting techniques can detect the presence of virus in plants, the establishment of disease cause requires identifying a direct association between a specific virus and the evident disease. Virome information can facilitate the introgression of viral resistance genes in molecular breeding. The review also presents newly sensitive detection techniques that can replace more costly quantitative reverse transcription polymerase chain reaction (qRT-PCR) diagnostic testing. A number of different digital disease assessment and phenotyping techniques are now available to save time and cost in breeding scores.

The use of traditional methods to increase genetic diversity and breed for improved yield and fiber quality is time-consuming, and the cost is high. Modern molecular technologies facilitate the identification of molecular markers and have thus emerged as new approaches in plant breeding. A team from Uzbekistan presented a review and analysis of the applications of molecular markers in the breeding and development of cotton germplasm (Kushanov et al.). Marker-based approaches include marker-assisted backcross selection, marker-assisted recurrent selection, marker-assisted gene pyramiding, and genomic selection. RNA-based sequencing technologies and transcriptome-wide association studies have also emerged in association studies of some important fiber traits. These new techniques may be appealing in future cotton breeding. Conventional breeding methods could be made more efficient by employing chromosome substitution lines to facilitate interspecific introgression.

Ratoon cultivation of cotton is a practice that could save cost, extend germplasm utilization time, and maintain male-sterile lines in hybrid cotton production. The review by Zhang et al. introduced the background of the perennial conservation of *Gossypium* species and gene pools and the genome assignment of the perennial species; discussed strategies for collecting, conserving, and characterizing perennial germplasm; and described the roles of ratoon cultivation in breeding. The review summarized and analyzed the different perennial cropping methods for ratoon cotton, the key measures for high yield of ratoon cotton, and the various applications of ratoon cultivation in cotton production.

## Original research

### Marker-assisted selection

Marker-assisted selection was studied in a breeding program by selection of superior fiber quality and agronomy traits using SSR markers (Darmanov et al.). Selections by the combined phenotypes (superior fiber quality) and marker genotypes (homozygous genotypes of Microsatellite (SSR) markers QTL) were applied in a backcross population. Two cultivars derived from the BC5F5 generation were developed with improved fiber strength and fiber length based on marker-assisted selections.

### Sequencing tools as molecular markers

In one study, the transcriptome, an RNA-based biotechnology, was used to analyze differentially expressed genes between parents with contrasting fiber quality (Jiang et al.). In another study, transcriptome analysis was used to reveal differential expression between 2,4-D–susceptible (TM-1) and tolerant plants (CS-B15sh) derived from chromosome substitution lines (Perez et al.). Components of the 2,4-D/auxin response pathway were identified as upregulated, with 3-fold higher expression in TM1 in contrast to CS-B15sh plants. Genes associated with herbicide metabolism also had 2-fold increased expression in CS-B15sh, which suggests the potential molecular basis of 2,4-D tolerance.

The sequencing-based marker single nucleotide polymorphism (SNP) has become a popular system in molecular marker analysis, with applications in cotton breeding. Two marker systems, genotype-by-sequencing (GBS) and SNP markers, were used in 250 recombinant inbred lines (RILs) and mapped 25 QTLs for the micronaire fiber trait (Pei et al.). Between two stable such QTLs, 338 genes were identified and 8 candidate genes were detected via differential expression between the two parents. In a similar study, 250 backcross inbred lines (BILs) derived from a cross between upland cotton and *G. barbadense* were screened with SNP markers for seed size and seed shape (Wu et al.). A total of 49 QTLs were identified explaining large variations of phenotypes. Further physiological analysis, genome sequencing, and gene expression analysis revealed five genes encoding mechanism-related starch synthase, which indicates possible candidate genes for seed size and shape. Another interesting study analyzed 181 intra-specific RILs by SNP markers for fiber quality under water stress (Boopath et al.). Fifty-three QTLs were detected for morphological and agronomic traits under water stress, with nine of them identified as major QTLs. Further analysis revealed putative candidate genes associated with water stress in the QTL hotspot on chromosome 22.

### New methods for disease resistance

The appropriate field evaluation method for resistance to FOV4 is critical in cotton disease resistance breeding. A study was conducted to investigate the effects of genotype, planting date, and inoculum density on disease progression (Zhang et al.). This study identified favorable temperatures at different times during planting season for FOV4 infection and further analyzed disease progression curves in different genotypes at different planting dates and inoculation methods. It was concluded that the disease progression curves can be used to demonstrate ROV4 infection with differential planting dates and inoculation methods.

Leafroll dwarf virus (CLRDV) is a disease detected in cotton worldwide. The detection of this disease is mainly based on RT-PCR, which is time-consuming and high-cost. A diagnostic method was developed using an enzyme-linked immunosorbent assay (ELISA) test (Hoffman et al.). This test used peptides based on the coat protein to produce polyclonal and monoclonal antibodies, which were used as a “double antibody sandwich” method for the ELISA test. The newly developed diagnostic method was found sensitive to CLRDV in cotton and weeds.

### Biotic and abiotic stresses: Mapping, resistance genes, and physiological traits

An RIL population derived from parents with differential levels of resistance to Race 7 of *F. wilt* was analyzed using SNP markers to study the genetic basis of resistance in cotton (Han et al.). Nine QTLs were identified for the Race 7 resistance. The gene expression study identified a candidate gene encoding calmodulin protein, and suppression of this gene lead to increased disease damage in plants. Aquaporins (AQPs) have been known for their role in water transport across cell membranes and thus in response to osmotic stress. Gao et al. identified a number of candidate genes in *Gossypium hirsutum, G. arboretum*, and *G. raimondii* (Guo et al.). Further gene expression studies confirmed a high expression of plasma intrinsic protein, *GhPIPs*, in *G. hirsutum*. These gene-silenced cotton plants showed damages in the chlorophyll and changed enzyme activities under salt stress. Furthermore, the overexpressed plants showed reduction of H_2_O_2_ under salt stress. The authors concluded that *GhPIPs* play positive regulatory roles.

The roles of sucrose:sucrose 1-fructosyltransferase (1-SST) in drought tolerance have been documented, but how the 1-SST–enhanced plants would perform in fields under stress was unknown. Liu et al. investigated ectopic expression in 1-SST–transformed cotton plants under drought stress and detected increased sugar, proline, and water contents. The yield loss under stress was reduced by 20% in the transgenic plants. This study confirmed the roles of sucrose 1-fructosyltransferase gene in drought tolerance at field level.

In another study, 181 RILs derived from intraspecific cross in upland cotton were compared between irrigated and limited water treatments and mapped using SNP markers (Boopathi et al.). Fifty-three QTLs were detected controlling morphological and agronomic traits under stress. One QTL hotspot was identified on chromosome 22 with a span length of 89.4 cM with 7 major QTLs for sympodial branch trait.

Limiting transpiration trait (TR_lim_) in plants under water stress would be ideal for alleviating yield loss under drought and saving water. Broughton and Conaty (Broughton and Conaty) studied cotton transpiration and yield under differential vapor pressure deficit (VPD). The difference of the limited transpiration trait was identified among Australian genotypes. A subsequent analysis revealed that this trait was not significant for reducing water use because of the negating effect due to increased transpiration rate at lower VPD environment in TR_lim_ cotton. The results suggested that this factor should be considered in future breeding for selecting genotypes under limited water stress.

### Other new tools for breeding

A comprehensive database was developed to demonstrate genomic variations and genome-wide associations in cotton (Peng et al.). This database has four modules for information including (1) genomics for locating the genomic position of the targeting sequences, (2) variations for identified polymorphic SNP and InDels, (3) genetics for detailed genome-wide association studies (GWAS) information, and (4) the capability to exhibit the genetic diversity of more than 3,000 sequenced tetraploid cotton genotypes. The database is available online for cotton researchers worldwide.

## Author contributions

LZ conceived the idea and coordinated the Research Topic. LZ, IW, and FB co-edited the Research Topic. All authors contributed to this article and approved the submitted version.

## Funding

LZ acknowledges financial support by USDA-ARS, Project No. 6066-21000-052-00D. FB was supported by the Arkansas Agriculture Experiment Station, Project No. 2658. IW acknowledges financial support by Cotton Breeding Australia, a joint venture between CSIRO and Cotton Seed Distributors Ltd.

## Conflict of interest

This study received funding from Cotton Seed Distributors Ltd. The funder was not involved in the study design, collection, analysis, interpretation of data, the writing of this article or the decision to submit it for publication. Author IW was employed by CSIRO, Agriculture and Food. The remaining authors declare that the research was conducted in the absence of any commercial or financial relationships that could be construed as a potential conflict of interest.

## Publisher's note

All claims expressed in this article are solely those of the authors and do not necessarily represent those of their affiliated organizations, or those of the publisher, the editors and the reviewers. Any product that may be evaluated in this article, or claim that may be made by its manufacturer, is not guaranteed or endorsed by the publisher.

